# How asthmatic patients use digital media looking for asthma information?

**DOI:** 10.1186/1939-4551-8-S1-A95

**Published:** 2015-04-08

**Authors:** Herberto Jose Chong Neto, Ivan Cherrez Ojeda, Juan Carlos Calderón Soriano, Nelson Rosario Filho, Marilyn Urrutia Pereira, Dirceu Sole

**Affiliations:** 1 Federal University of Parana, Brazil; 2 Respiralab, Hospital Kennedy, Ecuador; 3 Brazilian Sociaty, Brazil

## Background

The aim of this study was to verify how asthmatic patients use digital media looking for asthma information.

## Methods

Cross-sectional study using a standardized written questionnaire: How frequently do you use the social media? Do you use any social media to obtain asthma information? Would you like to receive asthma risk factors attacks and medication use orientation by social media? Have you interest to ask a doctor about asthma information using social media? The instrument was applied in Brazil (Curitiba, Uruguaiana and São Paulo) and Ecuator (Guayaquil). Patients or parents of children were invited to answer the questionnaire.

## Results

One hundred and eighty two patients or parents answered the questionnaire: 49.5% were males, median age 10 years (range 1 to 86) and a median disease time of 5 years (range 0 to 47). All patients were using asthma control medication; Facebook is the main social media used (50%). One hundred thirty six (76.4%) have no or limited access to the Web. Internet was the first choice (25.6%) of patients to obtain information about asthma. SMS was chosen as preferred digital media (66.7% and 64.7%, respectively) to receive asthma risk factors attacks, medication use recording and tool to ask a doctor. Fourty five (27.6%) had smartphone.

## Conclusions

Patients like to use social media to obtain asthma information, but access needs to be widely available.

